# Associations between post-traumatic stress symptoms and quality of life among psychiatric healthcare personnel in China during the COVID-19 pandemic: A network approach

**DOI:** 10.3389/fpsyt.2023.975443

**Published:** 2023-02-15

**Authors:** Yan-Jie Zhao, Cheng Zhang, Tong Guo, Sha Sha, Zhaohui Su, Teris Cheung, Todd Jackson, Feng-Rong An, Yu-Tao Xiang

**Affiliations:** ^1^Beijing Key Laboratory of Mental Disorders, Beijing Anding Hospital, The National Clinical Research Center for Mental Disorders, The Advanced Innovation Center for Human Brain Protection, School of Mental Health, Capital Medical University, Beijing, China; ^2^Yong Ding Lu Outpatient Department, Jingnan Medical Area, Chinese PLA General Hospital, Beijing, China; ^3^School of Public Health, Southeast University, Nanjing, China; ^4^School of Nursing, Hong Kong Polytechnic University, Hong Kong, Hong Kong SAR, China; ^5^Department of Psychology, University of Macau, Taipa, Macao SAR, China; ^6^Unit of Psychiatry, Department of Public Health and Medicinal Administration, Institute of Translational Medicine, Faculty of Health Sciences, University of Macau, Taipa, Macao SAR, China; ^7^Centre for Cognitive and Brain Sciences, University of Macau, Taipa, Macao SAR, China

**Keywords:** post-traumatic stress symptoms, quality of life, psychiatric healthcare personnel, COVID-19 pandemic, network

## Abstract

**Background:**

Post-traumatic stress symptoms (PTSS) are commonly reported by psychiatric healthcare personnel during the coronavirus disease 2019 (COVID-19) pandemic and negatively affect quality of life (QOL). However, associations between PTSS and QOL at symptom level are not clear. This study examined the network structure of PTSS and its connection with QOL in psychiatric healthcare personnel during the COVID-19 pandemic.

**Methods:**

This cross-sectional study was carried out between March 15 and March 20, 2020 based on convenience sampling. Self-report measures including the 17-item Post-Traumatic Stress Disorder Checklist – Civilian version (PCL-C) and World Health Organization Quality of Life Questionnaire - Brief Version (WHOQOL-BREF) were used to measure PTSS and global QOL, respectively. Network analysis was used to investigate the central symptoms of PTSS and pattern of connections between PTSS and QOL. An undirected network was constructed using an extended Bayesian Information Criterion (EBIC) model, while a directed network was established based on the Triangulated Maximally Filtered Graph (TMFG) method.

**Results:**

Altogether, 10,516 psychiatric healthcare personnel completed the assessment. “Avoidance of thoughts” (PTSS-6), “Avoidance of reminders” (PTSS-7), and “emotionally numb” (PTSS-11) were the most central symptoms in the PTSS community, all of which were in the *Avoidance and Numbing* domain. Key bridge symptoms connecting PTSS and QOL were “Sleep disturbances” (PTSS-13), “Irritability” (PTSS-14) and “Difficulty concentrating” (PTSS-15), all of which were within the *Hyperarousal* domain.

**Conclusion:**

In this sample, the most prominent PTSS symptoms reflected avoidance while symptoms of hyper-arousal had the strongest links with QOL. As such, these symptom clusters are potentially useful targets for interventions to improve PTSS and QOL among healthcare personnel at work under pandemic conditions.

## 1. Introduction

Since the coronavirus disease 2019 (COVID-19) was first reported in China at the end of 2019 and a pandemic was declared by the World Health Organization (WHO) in March 2020 (1), more than 200 countries and territories have been affected (2–4). As of December 2022, there have been over 659 million COVID-19 cases globally and over 6.6 million deaths, with the estimated mortality rate of 1.0% (2). In China, more than 400 thousands people had been diagnosed with COVID-19 by December, 2022 (5). The mortality rate caused by COVID-19 in China (1.2%) is similar with the global mortality rate. Due to the fast transmission of the novel virus, a relatively high death rate, the lack of specific medications and strict public health measures (e.g., suspension of public transportation, quarantine, and school closure) during early stages of the pandemic, many sub-populations suffered from mental health problems including depression, anxiety, insomnia, distress, and post-traumatic stress symptoms (PTSS) (6–9).

A previous meta-analysis revealed that 24, 23, 16 and 25% of all sub-populations reported depressive and anxiety symptoms, distress and PTSS, respectively during the COVID-19 pandemic (9). Of the psychological sequelae noted above, the prevalence of PTSS was highest. Healthcare professionals have had a higher prevalence of PTSS than the general population during the COVID-19 pandemic (28% vs. 19%), a difference that underscores how health professionals have been a high-risk population for PTSS during the pandemic (9).

PTSS comprises a host of psychiatric symptoms triggered by a traumatic event such as warfare, traffic collisions or other threats on a person’s life (10–12). PTSS includes disturbing thoughts, feelings, or dreams related to traumatic events, mental or physical distress associated with trauma-related cues, attempts to avoid trauma-related cues, alterations in the way one thinks and feels, and an increase in fight-or-flight responses (10, 13). Negative consequences of PTSS include psychiatric comorbidities, impaired functioning and lowered quality of life (QOL) (14–17).

The epidemiology of PTSS during outbreaks of acute infectious diseases has been examined previously (18–21). For example, one meta-analysis found that 17% of the general population experienced PTSS during the SARS outbreak while the corresponding figure during the COVID-19 pandemic was 25% (9). Recent studies found that up to 70% of healthcare professionals experienced COVID-19-related PTSS, due mainly to heavy clinical workloads and the fear of being infected (22). Apart from frontline health professionals, related populations including medical students who completed internships in hospital wards were also at risk for PTSS during early COVID-19 pandemic stages (23–25).

Similar to health professionals working in intensive care units or emergency departments (18, 19), psychiatric healthcare personnel have been susceptible to experiencing PTSS during the COVID-19 pandemic. In early stages of the pandemic, several nosocomial infections were reported in psychiatric hospitals and over 300 psychiatric inpatients and healthcare personnel were infected with COVID-19 (26, 27) because of significant challenges in patient management and deficient pandemic control measures such as protective equipment shortages, inadequate training in public health emergency responses, and increased vulnerability of psychiatric inpatients to infectious diseases (28). Under such circumstances, psychiatric healthcare personnel often suffered from overwhelming workplace-related stress and were more prone to have COVID-19-related PTSS (28, 29).

A common limitation of studies on PTSS during the COVID-19 pandemic has been an over-reliance upon traditional statistical approaches (e.g., a latent factor approach) based on mean scale scores from measures of PTSS and associated risk factors (30). Key premises underlying latent factor approaches are that all individual symptoms of a disorder are dependent on each other and are typically given equal weights toward calculating total scores; as a result, mutual associations among symptoms cannot be explored or adjusted for (30, 31). However, individual PTSS symptoms such as avoidance, numbing and irritability often exhibit robust associations with each other compared to other PTSS symptoms (32, 33). These data highlight how traditional statistical approaches can fail to capture complexity, nuances or dynamics between individual symptoms (34, 35).

Alternatively, network analysis has emerged as a novel approach to articulating links between psychiatric symptoms (36). Network analysis provides a better understanding of the most prominent symptoms of particular syndromes as well as the nature and strength of interconnections between individual symptoms and associations with other experiences such as QOL (36–39).

To date, two studies have explored the network structure of PTSS in the general population during the COVID-19 pandemic (40, 41); self-destructive/reckless behavior and internal avoidance were key PTSS symptoms. However, the network structure of PTSS and its connections with the widely used health outcome of QOL among psychiatric healthcare personnel remain unknown. Understanding the most prominent PTSS symptoms among frontline healthcare providers dealing with direct effects of the COVID-19 pandemic as well as symptom-level associations between PTSS and QOL is important for adopting effective preventive measures and treatments to reduce negative consequences caused by PTSS in this population. Therefore, this study examined the network structure of PTSS and its connection with QOL among psychiatric healthcare personnel during the COVID-19 pandemic.

## 2. Materials and methods

### 2.1. Study setting and participants

This was a cross-sectional, snowball-sampled study conducted between March 15 and March 20, 2020, shortly after the COVID-19 outbreak first emerged in China (3). Data were collected using the Questionnaire Star program (Changsha Haoxing Information Technology Co., Ltd., Changsha, China). A Quick Response (QR) code linked to the invitation and assessment forms was distributed by panel members of the Chinese Society of Psychiatry, and Chinese Nursing Association Branch of Psychiatry to all public psychiatric hospitals in China with WeChat, a social communication application with around 1.2 billion active monthly users in China (42) that is also used in continuing education projects of public psychiatric hospitals. To be eligible, participants needed to meet the following inclusion criteria: (1) aged 18 years or older; (2) frontline psychiatric healthcare personnel, including psychiatrists, nurses and nursing assistants working in clinical settings during the COVID-19 outbreak; (3) able to read Chinese and understand the purpose and contents of the assessments; (4) provide electronic written informed consent. The study protocol was approved by the Institutional Review Board (IRB) of Beijing Anding Hospital, China.

### 2.2. Assessment tools

The presence and severity of COVID-19-related PTSS was assessed by the validated Chinese version of the 17-item Post-Traumatic Stress Disorder Checklist – Civilian version (PCL-C) (43–45). The PCL-C covers three domains: *Intrusion* (items 1–5), *Avoidance and numbing* (items 6–12), and *Hyperarousal* (items 13–17). Each PCL-C item is rated from 1 (not at all) to 5 (extremely), with a higher score indicating more severe PTSS. The PCL-C has been validated with good psychometric properties (45–47).

Global QOL (QOL hereafter) was assessed with the first two items of the World Health Organization Quality of Life Questionnaire - brief version (WHOQOL-BREF). In these two items, participants were asked how they would rate their overall QOL and general health status based on item anchors ranging from 1 to 5; higher scores represented better reported QOL (48, 49). The Chinese version of WHOQOL has been validated in Chinese samples with good psychometric properties (48).

### 2.3. Data analysis

An undirected network model was constructed using the extended Bayesian Information Criterion (EBIC) model graphical least absolute shrinkage and selection operator (gLASSO) with a non-paranormal transformation (50, 51); thicker edges indicated stronger relationships between individual symptoms or nodes. The importance of nodes was evaluated using network centrality indices including strength and bridge strength. Strength is defined as the sum of absolute weights of the edge connecting a certain node to all other nodes (52). Bridge strength is defined as the sum of the absolute value of all edges that exist between node A and all nodes that are not in the same cluster as node A (53).

A directed network model was established based on the Triangulated Maximally Filtered Graph (TMFG) method (54). The TMFG method distinguishes “influencing” and “influenced” nodes based on the concept of node dependence (55). In a directed network figure, arrows indicate the direction of influence. Since the dependency matrix produced by the TMFG method is asymmetric (defining the two-way influences between node *i* and node *j* as D(*i, j*) and D(*j, i*)), asymmetric relations were calculated by retaining only stronger associations between D(*i, j*) and D(*j, i*), as recommended previously (53, 56).

Network stability was assessed using a 1,000-time case-dropping bootstrap method (57–59). In order to examine possible confounding effects of basic demographic factors, undirected networks were re-estimated after controlling for age and sex (34, 60). Visual inspection and Spearman correlation coefficient analyses for network centrality indices were used to evaluate effects of age and sex on the network structure.

All analyses were performed using *R*, version 4.1.0 (61) with the packages *bootnet* (59), *networktools* (62), *NetworkToolbox* (53), *psych* (63), *mgm* (64), and *qgraph* (65).

## 3. Results

### 3.1. Basic demographic characteristics

Altogether, 10,516 psychiatric healthcare personnel met the study entry criteria and completed the assessment. A majority of the participants was female (84.5%, 8,881/10,516). The sample ranged in age between 18 and 65 years, with an average age of 33.3 ± 8.4 years. The sample mean PCL-C score was 21.4 ± 6.4.

### 3.2. Network estimation and centrality measures

**Figure 1:**
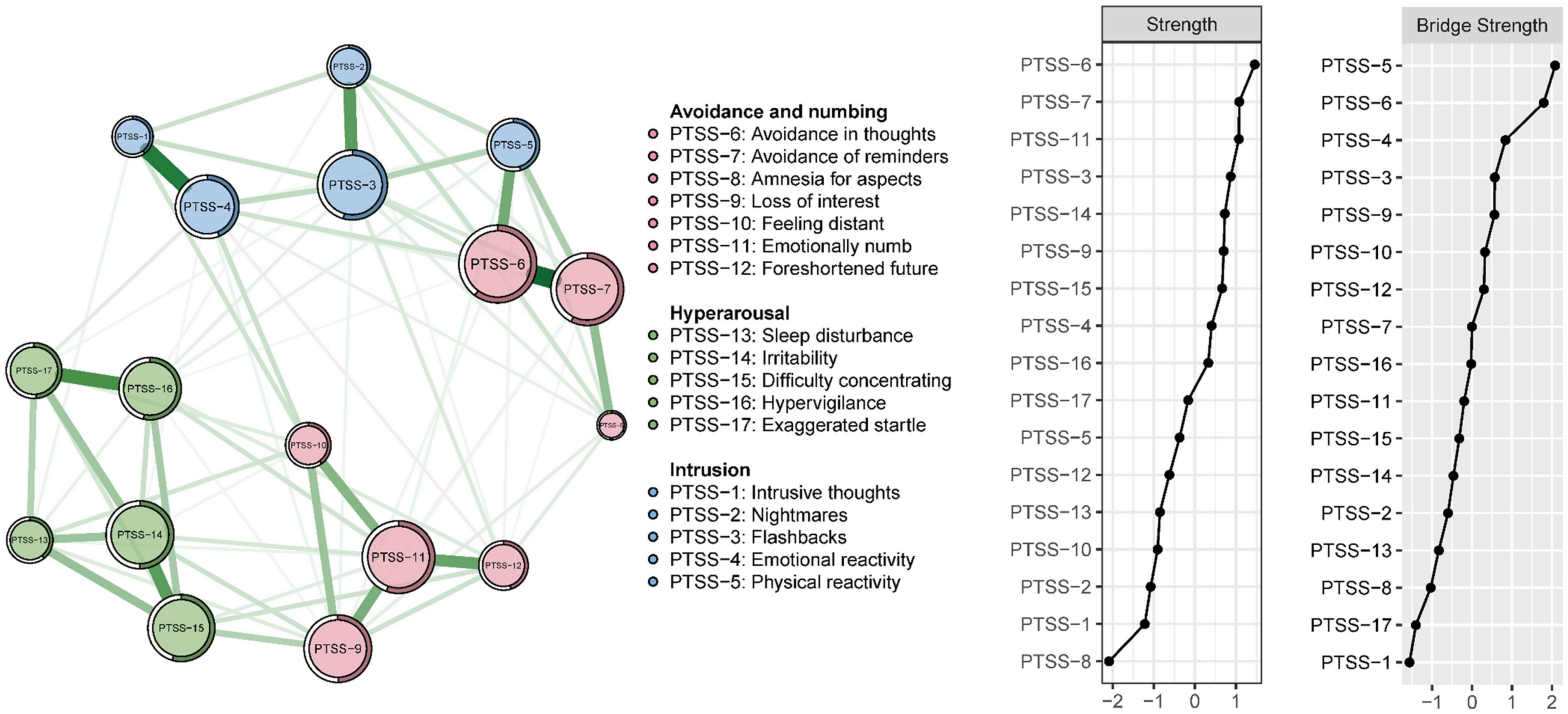
Network model of the PCL-C in the whole sample (*N* = 10,516) estimated by the Extended Bayesian Information Criterion graphical lasso (EBICglasso) method. The size of each node indicates the relative level of strength. Green edges indicate positive associations; red edges indicate negative associations. The values of strength and bridge strength were transformed into z-scores.

Means, standard deviations (*SD*), strengths, bridge strengths, predictability, skewness and kurtosis of PTSS symptoms are presented in Table 1. The network structure of PTSS among psychiatric healthcare personnel is displayed in Figure 1. “Avoidance of thoughts” (PTSS-6), “Avoidance of reminders” (PTSS-7), and “Emotionally numb” (PTSS-11) were the three most central nodes in PTSS network, all of which are members of the *Avoidance and numbing* domain.

**Table 1:** Mean, standard deviation, strength, bridge strength, predictability, skewness, and kurtosis of the PCL-C and QOL items (*N* = 10,516).

	Mean	SD	Strength	Bridge strength	Predictability	Strength*	Bridge strength*	Predictability*	Skewness	Kurtosis
PTSS-1	1.39	0.63	0.69	0.12	0.40	0.68	0	0.40	1.75	3.71
PTSS-2	1.09	0.35	0.71	0.24	0.43	0.68	0	0.43	4.63	26.89
PTSS-3	1.10	0.37	1.00	0.38	0.54	0.99	0	0.55	4.41	25.15
PTSS-4	1.33	0.61	0.93	0.41	0.44	0.87	0	0.44	2.08	5.34
PTSS-5	1.08	0.33	0.82	0.56	0.46	0.81	0	0.46	4.88	30.42
PTSS-6	1.10	0.36	1.09	0.52	0.60	1.09	0	0.60	4.37	23.97
PTSS-7	1.09	0.36	1.03	0.31	0.57	1.01	0	0.57	4.79	29.64
PTSS-8	1.13	0.39	0.56	0.18	0.32	0.56	0	0.32	3.73	17.77
PTSS-9	1.25	0.56	0.98	0.38	0.50	0.99	0.05	0.51	2.60	8.25
PTSS-10	1.35	0.68	0.74	0.35	0.40	0.73	0	0.41	2.29	6.01
PTSS-11	1.21	0.55	1.03	0.28	0.55	1.04	0.03	0.55	3.31	13.33
PTSS-12	1.15	0.49	0.78	0.34	0.45	0.68	0	0.45	4.15	20.97
PTSS-13	1.53	0.78	0.75	0.21	0.39	0.82	0.15	0.42	1.64	2.84
PTSS-14	1.46	0.74	0.98	0.25	0.50	0.99	0.09	0.51	1.89	4.22
PTSS-15	1.36	0.65	0.97	0.27	0.54	1.00	0.08	0.55	2.25	6.44
PTSS-16	1.26	0.60	0.92	0.31	0.54	0.92	0	0.54	2.85	9.68
PTSS-17	1.37	0.67	0.85	0.14	0.49	0.86	0.03	0.49	2.16	5.55
QOL-1	3.37	0.82	–	–	–	0.78	0.19	0.48	−0.49	0.16
QOL-2	3.27	0.93	–	–	–	0.82	0.23	0.49	−0.35	−0.62

Strength*, bridge strength*, and predictability* was calculated based on the network of PCL-C and QOL.

**Figure 2:**
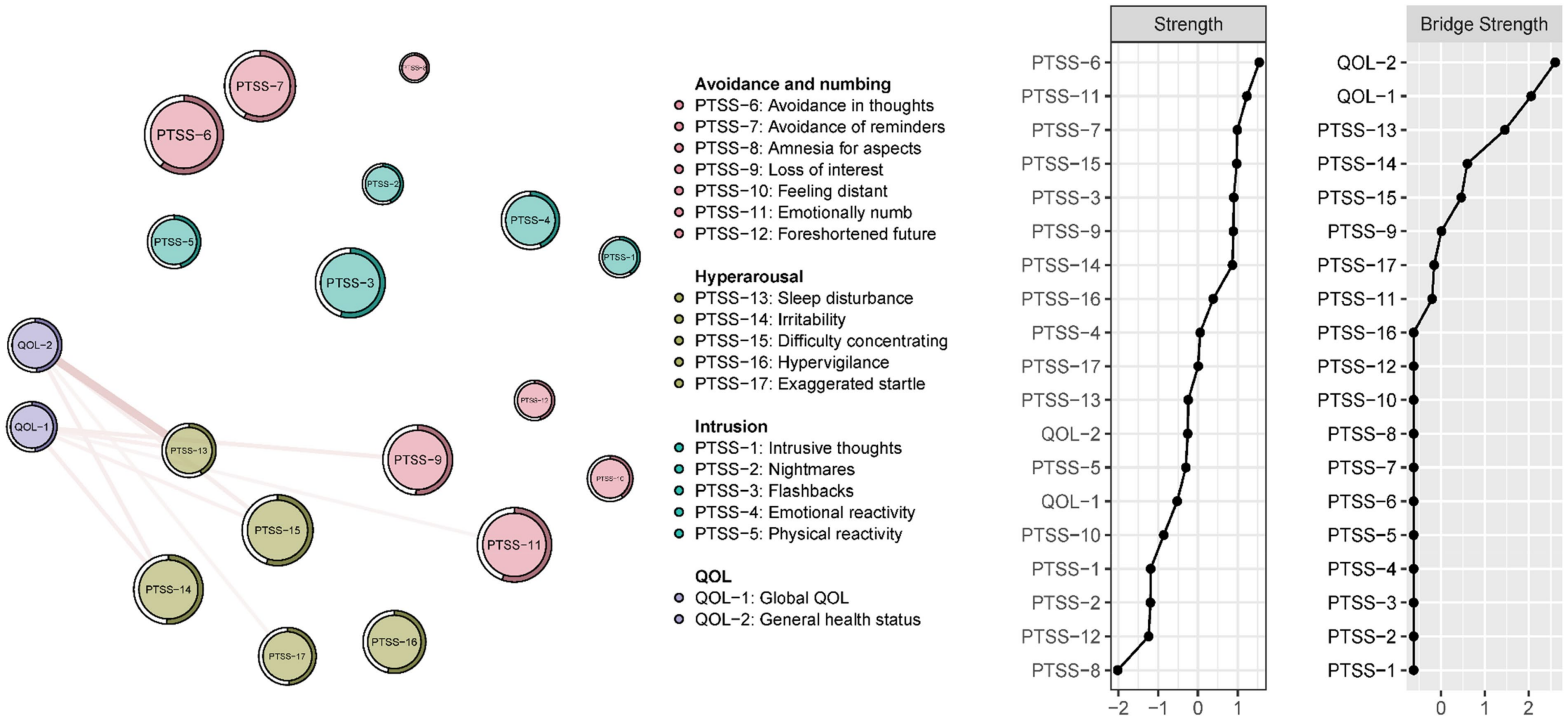
Bridge symptoms between the PCL-C and QOL items in the whole sample (N = 10,516). Green edges indicate positive associations; red edges indicate negative associations. The values of strength and bridge strength were transformed into z-scores.

The structure of bridge symptoms linking PTSS and QOL is shown in Figure 2. PTSS was negatively associated with QOL. Apart from the two nodes in the QOL cluster, “Sleep disturbances” (PTSS-13), “Irritability” (PTSS-14) and “Difficulty concentrating” (PTSS-15) were the three strongest bridge symptoms connecting PTSS and QOL. Of all interconnections between PTSS and QOL clusters, the edge between “sleep disturbances” and “general health status (QOL-2) had the strongest connection.

**Figure 3:**
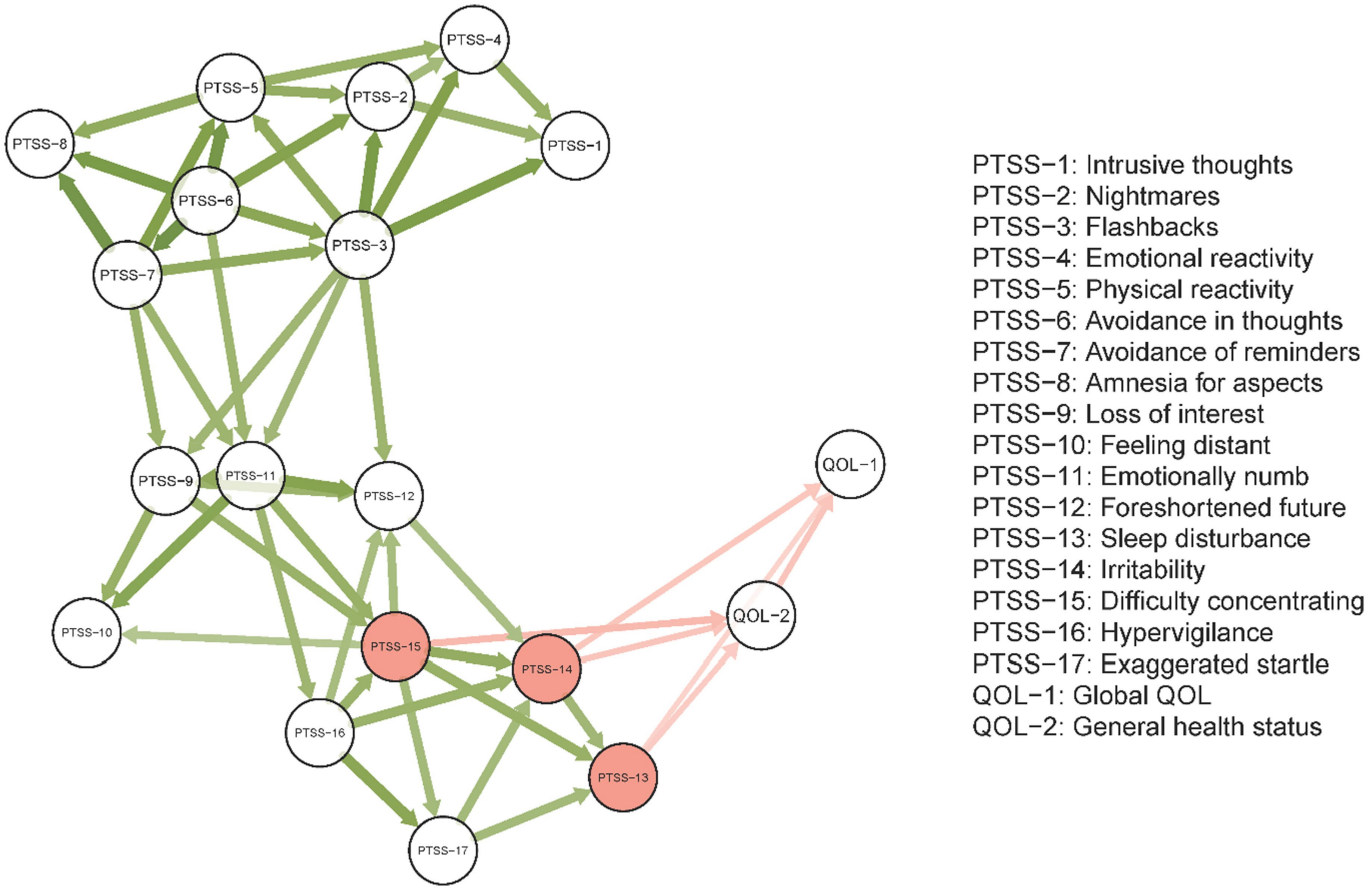
Directed network model of the PCL-C and QOL estimated by the Triangulated Maximally Filtered Graph (TMFG) method. Edge thickness indicates edge weight. Red nodes have the highest bridge strength in the undirected network. Arrows indicate the direction of influence. Defining the two-way influences between node *i* and node *j* as D(*i,* *j*) and D(*j,* *i*), only the greater link between D(*i, j*) and D(*j, i*) is retained.

The directed PTSS-QOL network model is displayed in Figure 3. The most prominent arrows between the two clusters were from PTSS-13, PTSS-14, and PTSS-15 to QOL-1 and QOL-2, paralleling key bridging edges identified in the undirected network. Based on the assumption that PTSS had “influencing” effects on QOL, these results indicated that psychiatric healthcare personnel with severe “Sleep disturbances” (PTSS-13), “Irritability” (PTSS-14) and “Difficulty concentrating” (PTSS-15) had lower QOL compared to healthcare personnel who experienced other PTSS symptoms.

### 3.3. Confounding effects of age and sex

**Figure 4:**
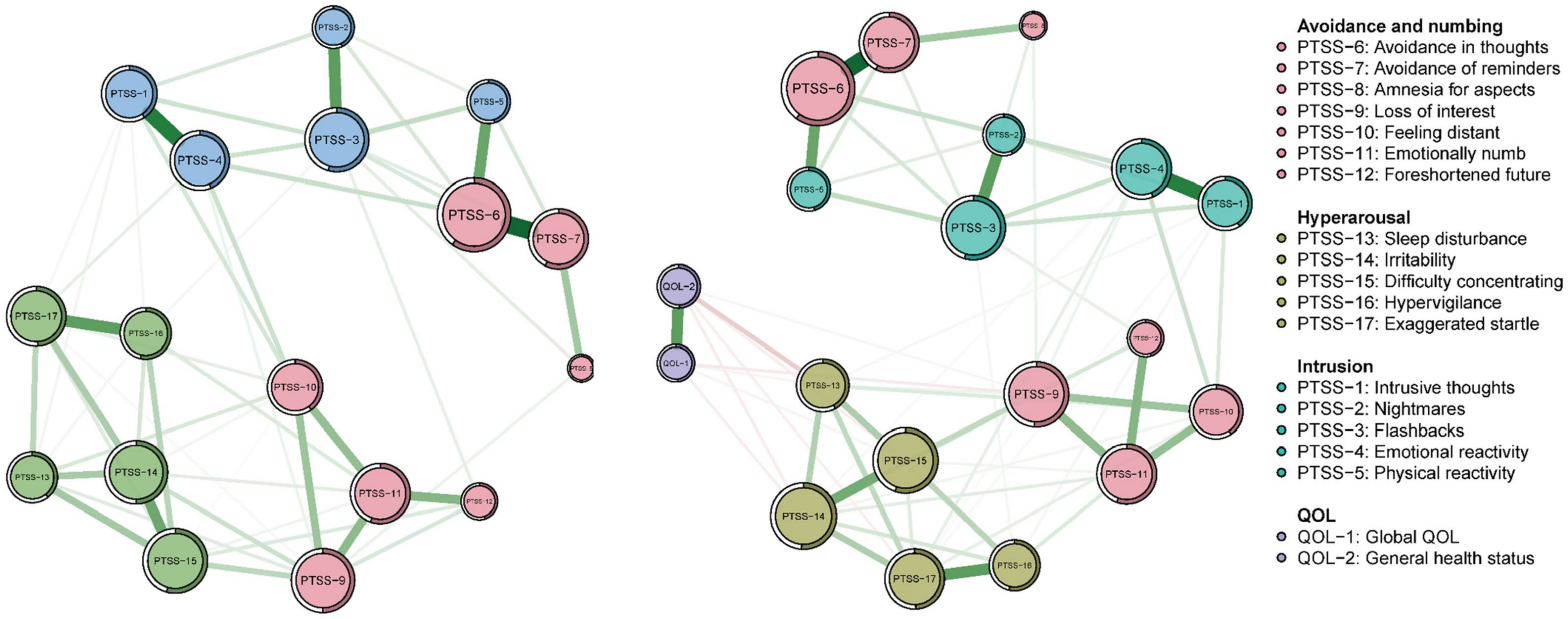
Network models in the whole sample (*N* = 10,516) estimated by the Extended Bayesian Information Criterion (EBIC) method after adjusting for age and sex. Network model of PCL-C after adjusting for covariates (**left**) and network model of PCL-C and QOL after adjusting for covariates (**right**). The size of each node indicates the relative level of strength. Green edges indicate positive associations; red edges indicate negative associations. For easy comparison with network graphs without adjusting for covariates, the layouts of panels followed the layouts of the two network graphs without adjusting for covariates (Figure 1 and Supplementary Figure S1).

Undirected network models, adjusted for age and sex (confounds hereinafter), are displayed in Figure 4. Visual inspection of these data indicated the network structures and connection patterns were similar before and after adjusting for sex and age. For PCL-C network models, Spearman correlation coefficients between initial (unadjusted) and subsequent (age- and sex-controlled) models for strength and bridge strength were 0.82 and 0.67, respectively (both *p* values were < 0.001). For the network model comprising PCL-C and QOL, Spearman correlation coefficients between initial (unadjusted) and subsequent (confound-controlled) models for strength and bridge strength were 0.84 and 0.96, respectively (both *p* values <0.001). These results indicated that age and sex did not have significant confounding effects on the two primary undirected networks.

### 3.4. Network stability

The case-dropping bootstrap procedure showed that the undirected PCL-C network and undirected PCL-C and QOL network values for strength and bridge strength remained stable after randomly dropping 75% of the sample (Supplementary Figure S2). *CS-Coefficients* for strength and bridge strength in the two networks were both 0.75, indicating that 75% of the sample could be dropped and network structures would remain stable.

## 4. Discussion

To our knowledge, this is the first study to identify the network structure of PTSS and its connections with QOL among psychiatric practitioners during the COVID-19 pandemic. “Avoidance in thoughts” (PTSS-6), “Avoidance of reminders” (PTSS-7), and “Emotionally numb” (PTSS-11) were the three most influential nodes in the network model, all of which are in the *Avoidance and numbing* domain of PTSS. This finding contrasts with the network structure of COVID-19-related PTSS found in a study of the general population wherein self-destructive/reckless behavior, emotional reactivity and nightmares were central symptoms (40). Inconsistencies between these models are likely due to different study samples (general population vs. psychiatric healthcare personnel) and possible demands upon psychiatric practitioners to serve others and inhibit from overt personal expressions of distress in their professional roles.

Previous studies have found that, apart from comorbid depression, anxiety and social phobia (32, 66), avoidance coping has strong associations with the development of chronic PTSD (67). In this study “Avoidance of thoughts” (PTSS-6) and “Avoidance of reminders” (PTSS-7) had the highest strengths in the network model of PTSS for psychiatric healthcare personnel. “Avoidance of thoughts” refers to avoiding thinking, talking or having feelings about stressful events (e.g., the COVID-19 pandemic), while “Avoidance of reminders” refers to avoiding activities or situations that would remind an individual of a stressful experience (43). Our findings underscored how many psychiatric healthcare personnel tended to cope during the COVID-19 pandemic by disengaging from relevant thoughts/reminders rather than engaging in them (40). These two items had relatively low mean scores but were the top-ranked centrality indices and support the notion that avoidance symptoms are not openly expressed but represent an important diagnostic indicator of PTSD (32, 68). It should be noted that although avoidant coping could provide short-term psychological protection and reduce distress of those affected by traumatic events (66), chronic avoidance over extended durations is considered to be maladaptive and contributes to sustained stress symptoms and impaired post-traumatic recovery (33, 69).

To highlight the importance of avoidance in PTSD symptom clusters, the Diagnostic and Statistical Manual of Mental Disorders - 5th edition (DSM-V) revised previous PTSD diagnostic criteria by separating the avoidance and numbing cluster into two independent clusters (10, 70). The DSM-V requires both avoidance and numbing symptoms for the diagnosis of PTSD (10), and is consistent with concomitant avoidance and numbing symptoms as the most prominent ones in this study.

As another key central symptom in the current network model, “emotional numbing” refers to having remarkably reduced ability to feel emotions (10), especially positive emotions such as tender, affectionate or loving feelings, accompanied by a lack of care or concern for oneself and others (43, 71). A previous study contended that “emotional numbing” is a unique symptom of PTSD that can be used to differentiate PTSD from major depressive disorder (MDD) despite substantial overlaps in symptomatology and comorbidity between these two disorders (72).

The high centrality of “emotional numbing” in the current PTSS network might be due to several factors. Frontline healthcare professionals are vulnerable to occupational burnout and fatigue due to heavy workloads during the pandemic (73, 74). Due to depleted physiological and psychological reserves in managing chronically heavy clinical workloads and possible personal COVID-19-related hyper-arousal symptoms, emotional resources of frontline psychiatric practitioners may become compromised and contribute to emotional numbing (75, 76). Additionally, emotional numbing can result from avoidance of traumatic triggers rather than exposure to traumatic events *per se*; avoidance of such triggers might lead, not only to reduced exposure to emotional provoking situations, but also to a “shut down” of affective system (75, 77, 78), per the central role of emotional numbing within the network model from this study. Finally, prospective studies have found that emotional numbing is related to increased future risk for the development of PTSD (33, 67) and depression (72).

As expected, bridging edges between PTSS and QOL indicated elevations within the PTSS community were associated with lower QOL, consistent with previous findings (79–81). “Sleep disturbances” (PTSS-13), “Irritability” (PTSS-14) and “Difficulty concentrating” (PTSS-15) were the most important bridging symptoms connecting the PTSS community and QOL in this sample. Sleep disturbances contribute to various negative outcomes such as impaired daily functioning (82), lowered career satisfaction (83) and emotional dysregulation (84), all of which could lower QOL (85, 86). “Irritability” refers to being easily provoked, irritated, or having uncontrolled outbursts of anger (87), and is a symptom in the *Hyper-arousal* domain of PTSS (10). In previous studies, irritability was associated with impaired QOL (88, 89), in line with our findings. One randomized control trial provided evidence that QOL could be improved through the treatment of irritability among autistic children (90), though extensions warrant consideration in psychiatric professionals. “Difficulty concentrating” implies an individual’s cognitive resources are overwhelmed by demands that exceed cognitive resources and is often accompanied by impairments in daily functioning and work performance (91–93) that could contribute to lower QOL (94, 95).

Strengths of this study included its large sample size of an understudied population (i.e., frontline healthcare workers in early stages of a global pandemic) and the use of network analysis to illuminate inter-relationships between particular PTSS symptoms and QOL. Several limitations should also be noted. First, although the TMFG method generated directional arrows between nodes of PTSS and QOL, associated findings must be interpreted in the context of a non-experimental cross-sectional study design. Directions of influence produced by the TMFG method represented more statistically probable “influencing” and “influenced” relationships among symptoms (54, 55) that provide empirically-grounded yet preliminary hypotheses that need to be tested *via* experimental studies and/or prospective research designs (96). Second, because only psychiatric practitioners were included, findings may not be generalized to related populations such as general health practitioners. Third, for logistical reasons, the mental health status of participants prior to the COVID-19 outbreak was not assessed so the presence and impact of pre-COVID-19 emotional problems on current PTSS and QoL could not be examined. Fourth, due to strictly enforced safety protocols in early stages of COVID-19 pandemic, assessments were limited to validated measures of PTSS and QOL rather in-person examination *via* structured clinical interviews. Fifth, following other studies conducted during the COVID-19 pandemic (74, 97), for logistical reasons, convenience sampling was used in this study. Hence, selection biases may have affected the data. Furthermore, most of the participants (84.5%) were women. This aligns with the actual gender distribution among healthcare personnel in China. For example, 74.4% of healthcare personnel in China during 2020 were female (98). Nonetheless, the proportion of women in our sample exceeded this national estimate. Finally, because there was no control sample in this study, comparisons of psychiatric healthcare professionals with responses from the general population could not be made.

In conclusion, among psychiatric healthcare professionals working during early stages of the COVID-19 pandemic, central PTSS included “Avoidance of thoughts” “Avoidance of reminders” and “Emotional numbness.” In addition, key bridge symptoms linking PTSS with QOL in this group included hyper-arousal domain facets, “Sleep disturbances,” “Irritability” and “Difficulty concentrating.” Based on these findings, this study suggests that PTSS symptoms of avoidance and hyper-arousal should be monitored among psychiatric healthcare professionals during the pandemic and serve as potentially important targets for prevention and treatment for those with PTSS in these circumstances.

## Data availability statement

The datasets presented in this article are not readily available because the Institutional Review Board (IRB) of Beijing Anding Hospital that approved the study prohibits the authors from making publicly available the research dataset of clinical studies. Requests to access the datasets should be directed to xyutly@gmail.com.

## Ethics statement

The studies involving human participants were reviewed and approved by the Institutional Review Board (IRB), Beijing Anding Hospital. The patients/participants provided their electronic written informed consent to participate in this study.

## Author contributions

F-RA and Y-TX: study design. Y-JZ, CZ, TG, SS, ZS, and F-RA: data collection, analysis, and interpretation. Y-JZ and Y-TX: drafting of the manuscript. TJ: critical revision of the manuscript. All authors: approval of the final version for publication.

## Funding

This study was supported by the Beijing Anding Hospital, Capital Medical University (No. KY296), Beijing Municipal Science and Technology Commission (Grant No. Z181100001718124), Beijing Talents Foundation (Grant No. 2017000021469G222), University of Macau (No. MYRG2019-00066-FHS), Scientific Research Common Program of Beijing Municipal Commission of Education (No. KM202010025011), and Beijing Municipal Science and Tech Commission (No. Z191100006619061).

## Conflict of interest

The authors declare that the research was conducted in the absence of any commercial or financial relationships that could be construed as a potential conflict of interest.

## Publisher’s note

All claims expressed in this article are solely those of the authors and do not necessarily represent those of their affiliated organizations, or those of the publisher, the editors and the reviewers. Any product that may be evaluated in this article, or claim that may be made by its manufacturer, is not guaranteed or endorsed by the publisher.
