# Supplementary material for: Associations between post-traumatic stress symptoms and quality of life among psychiatric healthcare personnel in China during the COVID-19 pandemic: A network approach
Source: Front Psychiatry. 2023 Feb 15;14:975443. doi: 10.3389/fpsyt.2023.975443 (PMC9975756; doi:10.3389/fpsyt.2023.975443)
Supplement: Supplementary file 1 [file Data_Sheet_1.pdf]

Supplementary Materials

Supplementary Table 1. Adjacency matrix of PCL-C items

Supplementary Table 2. Filtered dependency matrix of PCL-C and QOL

Supplementary Figure 1. Undirected network model for PCL-C and QOL in the total sample (N=10,516) estimated by the Extended Bayesian Information Criterion graphical lasso (EBICglasso) method

Supplementary Figure 2. Central stability by case-dropping bootstrap method

Supplementary Figure 3. Bootstrapped confidence intervals of edge weights in the network of PCL-C

Supplementary Figure 4. Estimation of edge weight differences by bootstrapped difference test in the network of PCL-C

Supplementary Figure 5. Estimation of node strength differences by bootstrapped difference test in the network of PCL-C

Supplementary Table 1. Adjacency matrix of PCL-C items

	PTSS-1	PTSS-2	PTSS-3	PTSS-4	PTSS-5	PTSS-6	PTSS-7	PTSS-8	PTSS-9	PTSS-10	PTSS-11	PTSS-12	PTSS-13	PTSS-14	PTSS-15	PTSS-16	PTSS-17
PTSS-1	0	0.09	0.10	0.38	0	0	0	0	0	0.09	0	0	0.03	0	0	0	0
PTSS-2	0.09	0	0.29	0	0.10	0.09	0.05	0.03	0	0	0	0.03	0	0	0	0.03	0
PTSS-3	0.10	0.29	0	0.11	0.13	0.08	0.07	0.05	0.04	0	0	0.06	0	0	0	0.04	0.03
PTSS-4	0.38	0	0.11	0	0.03	0.09	0.04	0.04	0.03	0.09	0	-0.04	0	0.02	0	0	0.05
PTSS-5	0	0.10	0.13	0.03	0	0.24	0.14	0.06	0	0	0.04	0.03	0	0	0	0.04	0
PTSS-6	0	0.09	0.08	0.09	0.24	0	0.45	0.08	0.03	0	0	0	0	0	0	0.02	0
PTSS-7	0	0.05	0.07	0.04	0.14	0.45	0	0.19	0.03	0	0.03	0.02	0	0	0	0	0
PTSS-8	0	0.03	0.05	0.04	0.06	0.08	0.19	0	0.05	0	0	0.05	0	0	0	0	0
PTSS-9	0	0	0.04	0.03	0	0.03	0.03	0.05	0	0.18	0.23	0.09	0.06	0.09	0.13	0.03	0
PTSS-10	0.09	0	0	0.09	0	0	0	0	0.18	0	0.21	0	0.08	0.03	0	0	0.06
PTSS-11	0	0	0	0	0.04	0	0.03	0	0.23	0.21	0	0.27	0.04	0.07	0.05	0.08	0
PTSS-12	0	0.03	0.06	-0.04	0.03	0	0.02	0.05	0.09	0	0.27	0	0	0.04	0.09	0.06	0
PTSS-13	0.03	0	0	0	0	0	0	0	0.06	0.08	0.04	0	0	0.18	0.18	0.04	0.14
PTSS-14	0	0	0	0.02	0	0	0	0	0.09	0.03	0.07	0.04	0.18	0	0.29	0.10	0.17
PTSS-15	0	0	0	0	0	0	0	0	0.13	0	0.05	0.09	0.18	0.29	0	0.15	0.08
PTSS-16	0	0.03	0.04	0	0.04	0.02	0	0	0.03	0	0.08	0.06	0.04	0.10	0.15	0	0.32
PTSS-17	0	0	0.03	0.05	0	0	0	0	0	0.06	0	0	0.14	0.17	0.08	0.32	0

Supplementary table 2. Filtered dependency matrix of PCL-C and QOL

	PTSS-1	PTSS-2	PTSS-3	PTSS-4	PTSS-5	PTSS-6	PTSS-7	PTSS-8	PTSS-9	PTSS-10	PTSS-11	PTSS-12	PTSS-13	PTSS-14	PTSS-15	PTSS-16	PTSS-17	QOL-1	QOL-2
PTSS-1	0	0.23	0.21	0.27	0	0	0	0	0	0	0	0	0	0	0	0	0	0	0
PTSS-2	0.31	0	0.30	0.28	0.27	0.26	0	0	0	0	0	0	0	0	0	0	0	0	0
PTSS-3	0.39	0.41	0	0.38	0.34	0.32	0.32	0	0.29	0	0.29	0.30	0	0	0	0	0	0	0
PTSS-4	0.32	0.24	0.24	0	0.23	0	0	0	0	0	0	0	0	0	0	0	0	0	0
PTSS-5	0	0.33	0.32	0.32	0	0.34	0.32	0.34	0	0	0	0	0	0	0	0	0	0	0
PTSS-6	0	0.37	0.36	0	0.42	0	0.44	0.41	0	0	0.30	0	0	0	0	0	0	0	0
PTSS-7	0	0	0.34	0	0.38	0.42	0	0.43	0.31	0	0.30	0	0	0	0	0	0	0	0
PTSS-8	0	0	0	0	0.22	0.21	0.22	0	0	0	0	0	0	0	0	0	0	0	0
PTSS-9	0	0	0.24	0	0	0	0.24	0	0	0.32	0.32	0.31	0	0	0.31	0	0	0	0
PTSS-10	0	0	0	0	0	0	0	0	0.24	0	0.25	0	0	0	0.21	0	0	0	0
PTSS-11	0	0	0.25	0	0	0.25	0.25	0	0.35	0.36	0	0.37	0	0	0.32	0.30	0	0	0
PTSS-12	0	0	0.21	0	0	0	0	0	0.25	0	0.27	0	0	0.26	0.26	0.24	0	0	0
PTSS-13	0	0	0	0	0	0	0	0	0	0	0	0	0	0.21	0.20	0	0.20	-0.13	-0.15
PTSS-14	0	0	0	0	0	0	0	0	0	0	0	0.23	0.29	0	0.29	0.25	0.27	-0.16	-0.17
PTSS-15	0	0	0	0	0	0	0	0	0.26	0.23	0.25	0.27	0.32	0.33	0	0.28	0.28	0	-0.20
PTSS-16	0	0	0	0	0	0	0	0	0	0	0.26	0.27	0	0.31	0.31	0	0.36	0	0
PTSS-17	0	0	0	0	0	0	0	0	0	0	0	0	0.28	0.28	0.26	0.29	0	0	0
QOL-1	0	0	0	0	0	0	0	0	0	0	0	0	0.12	0.11	0	0	0	0	-0.16
QOL-2	0	0	0	0	0	0	0	0	0	0	0	0	0.12	0.11	0.11	0	0	-0.17	0

Supplementary Figure 1. Undirected network model for the PCL-C and QOL in the whole sample (N=10,516) estimated by the Extended Bayesian Information Criterion graphical lasso (EBICglasso) method

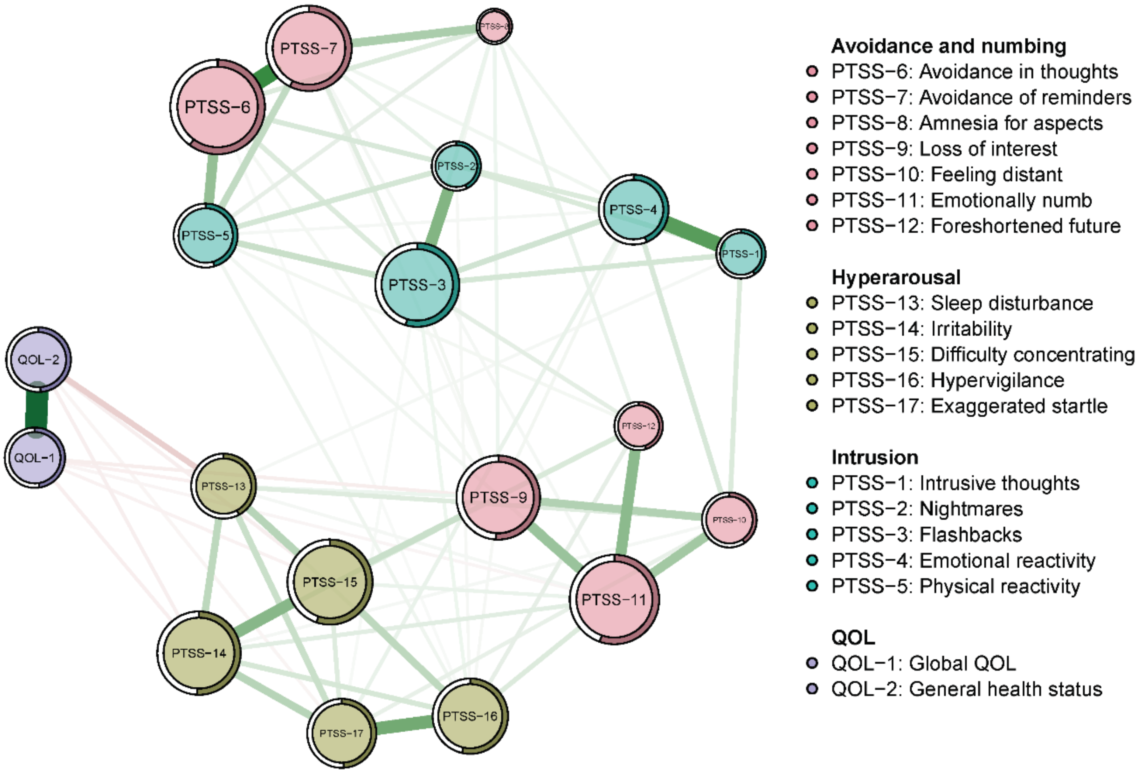

Figure legend: The size of each node indicates the relative level of strength. Green edges indicate positive associations; red edges indicate negative associations.

Supplementary Figure 2. Central stability by case-dropping bootstrap method

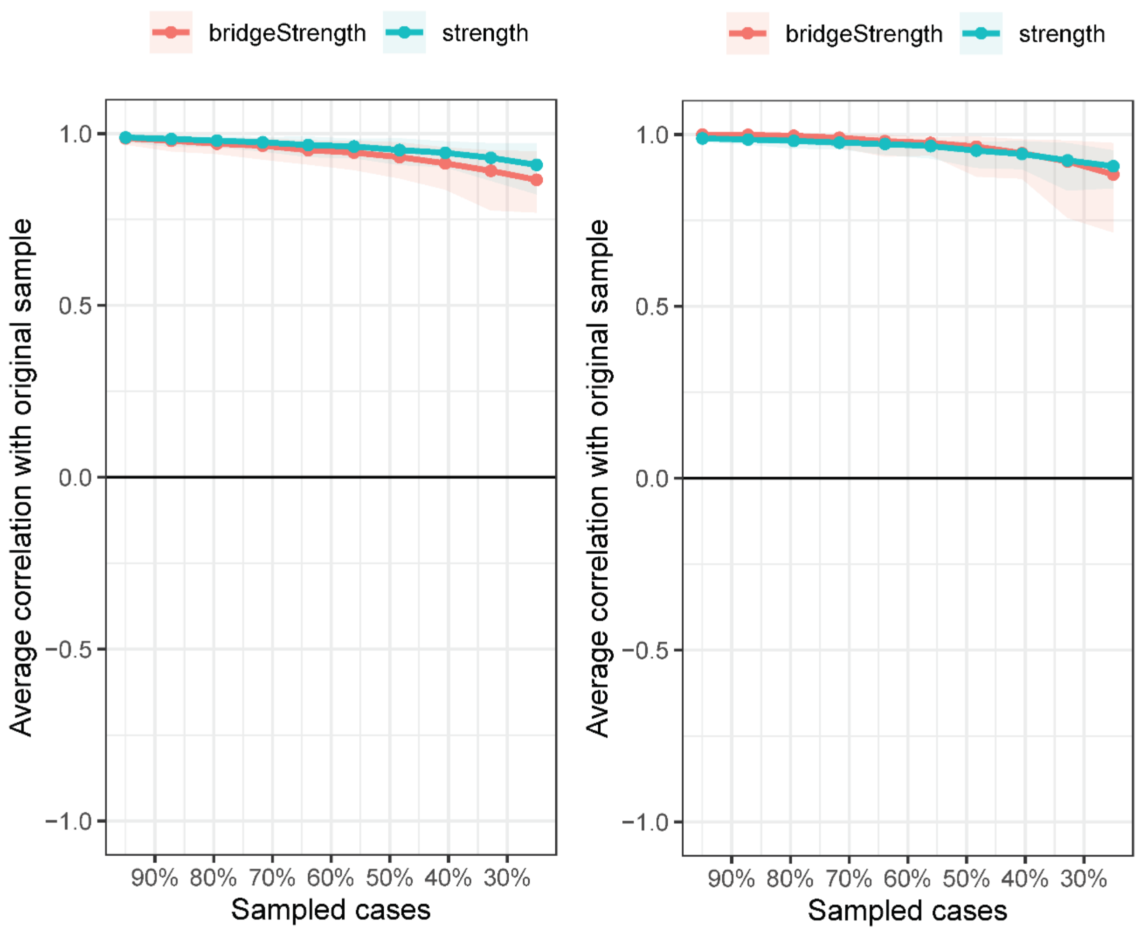

Figure legend: Central stability in the network of PCL-C (**left panel**) and in the network of PCL-C and QOL (**right panel**). The x-axis represents the percentage of cases of the original sample remained at each case-dropping subset. The y-axis represents the average of correlations between the centrality indexes from the original network and the re-estimated network after case-dropping procedure.

Supplementary Figure 3. Bootstrapped confidence intervals of edge weights in the network of PCL-C

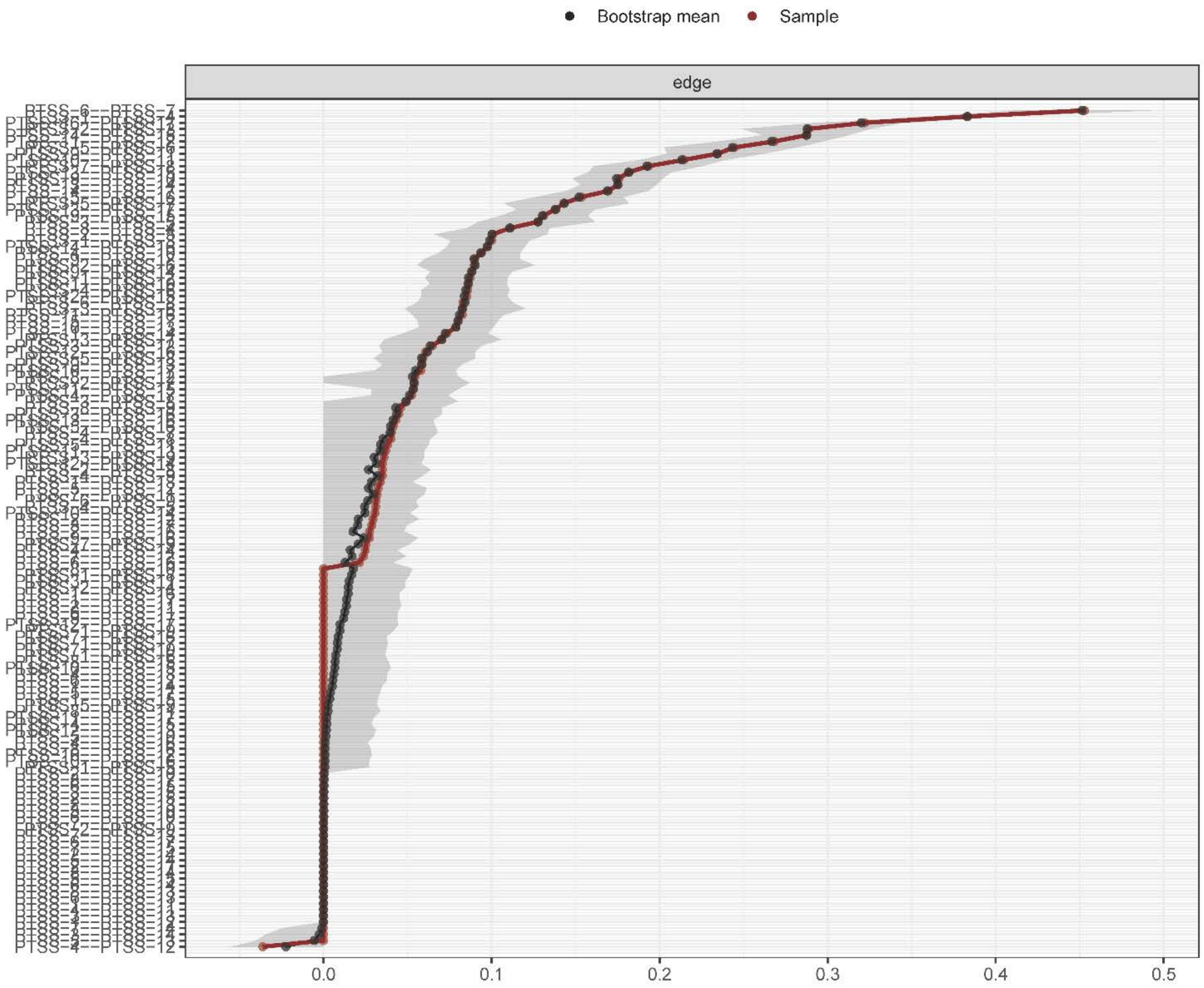

Figure legend: The red dots indicate the estimated values of each edge weight, ordered from the highest to the lowest value. The black dots indicate the observed value of each edge weight. The gray area represents the 95% Confidence Intervals of edge weights, estimated with the non-parametric bootstrap procedure. Wide intervals indicate lower stability and narrow intervals indicate higher stability.

Supplementary Figure 4. Estimation of edge weight differences by bootstrapped difference test in the network of PCL-C

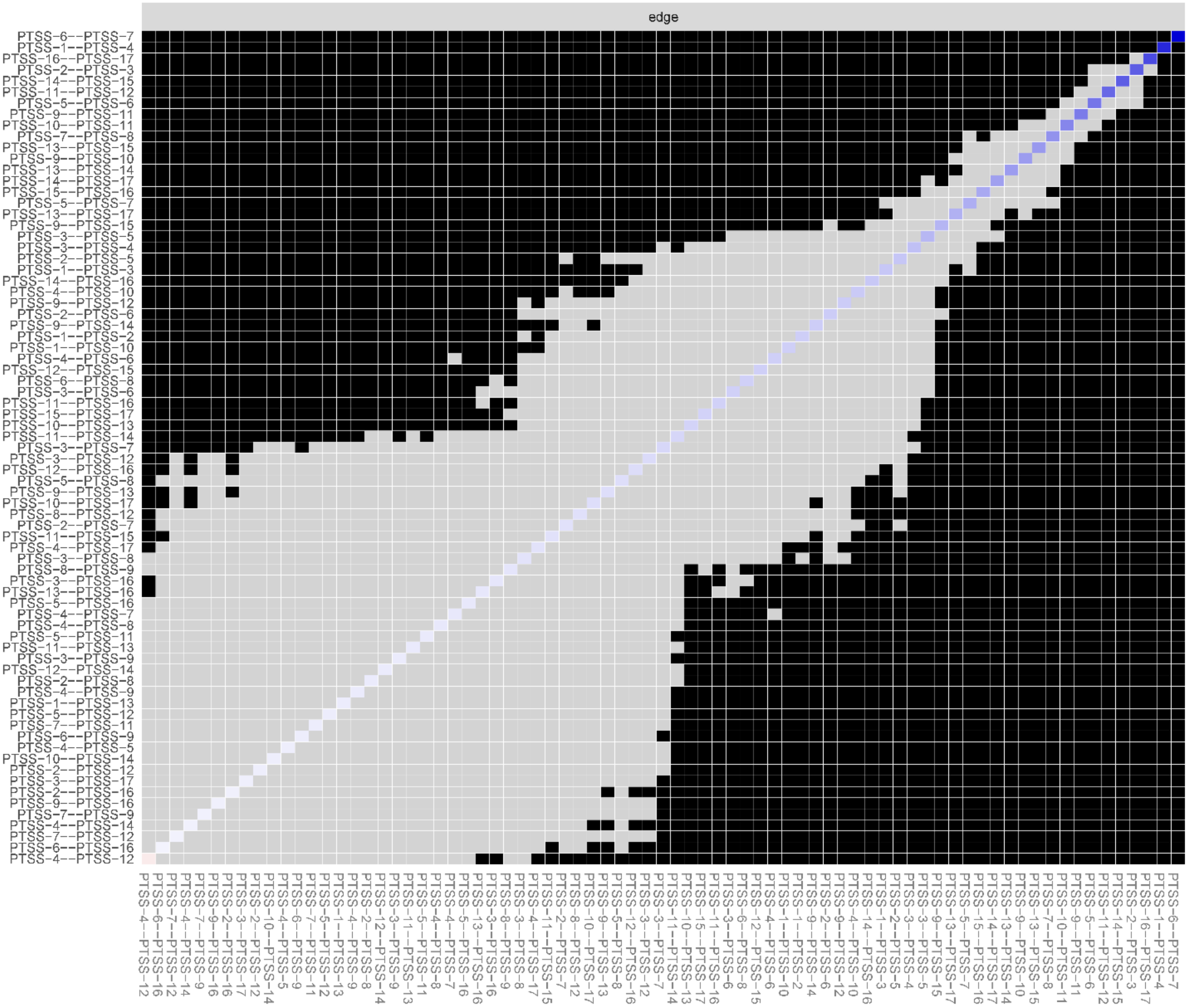

Figure legend: Gray boxes indicate edges that do not differ from one-another significantly. Black boxes represent edges with significant difference from one another (significance level = 0.05). Blue boxes in the edge-weight plot correspond to positive correlations and red boxes to negative correlations.

Supplementary Figure 5. Estimation of node strength differences by bootstrapped difference test in the network of PCL-C

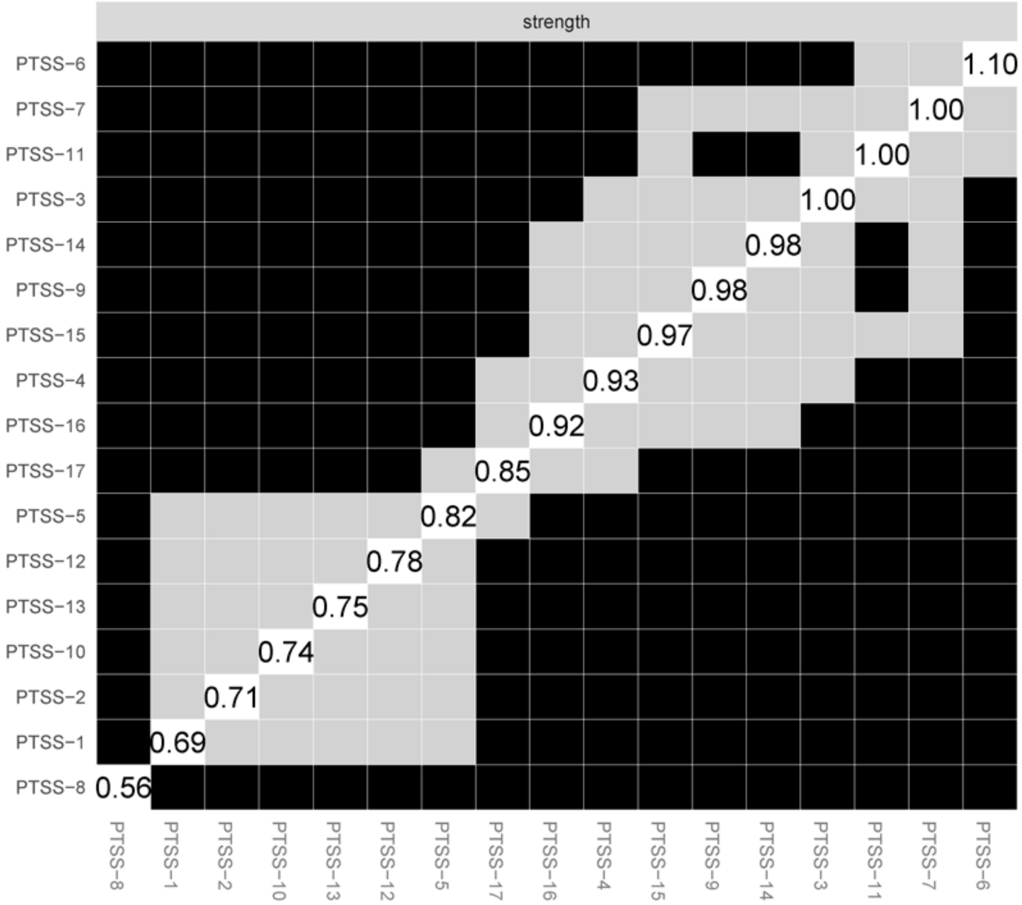

Figure legend: Gray boxes indicate nodes that do not differ from one-another significantly. Black boxes represent nodes that differ significantly from one another (significance level = 0.05). White boxes show the values of node strength.
